# Flupentixol tartrate

**DOI:** 10.1107/S1600536814001536

**Published:** 2014-01-29

**Authors:** Thammarse S. Yamuna, Manpreet Kaur, Brian J. Anderson, Jerry P. Jasinski, H.S. Yathirajan

**Affiliations:** a Department of Studies in Chemistry, University of Mysore, Manasagangotri, Mysore 570 006, India; b Department of Chemistry, Keene State College, 229 Main Street, Keene, NH 03435-2001, USA

## Abstract

In the title salt, C_23_H_26_F_3_N_2_OS^+^·C_4_H_5_O_6_^−^ [systematic name: 1-(2-hy­droxy­eth­yl)-4-[3-(2-(tri­fluoro­meth­yl)thioxanthen-9-yl­idene)prop­yl]piperazin-1-ium 3-carb­oxy-2,3-di­hydroxy­pro­pion­ate], the monoprotonated piperazine ring in the cation adopts a chair conformation, while the thio­pyran ring of the thioxanthene group has a boat conformation. The dihedral angle between the mean planes of the two outer aromatic rings of the thioxanthene groups is 31.6 (2)°. In the crystal, the cations and anions are linked *via* O—H⋯O, N—H⋯O, O—H⋯N and C—H⋯O hydrogen bonds, forming chains propagating along [100]. In addition, *R* ^2^_2_(7), *R* ^2^_2_(11), *R* ^2^_2_(10) and *R* ^2^_2_(12) graph-set ring motifs involving the anions, and *R* ^2^_2_(9) graph-set ring motifs involving both the cations and anions are observed. The three F atoms of the tri­fluoro­methyl group are disordered over two sets of sites and the individual atoms were refined with occupancy ratios of 0.54 (6):0.46 (6), 0.72 (2):0.28 (2) and 0.67 (3):0.33 (3).

## Related literature   

For general background and the pharmacological properties of flupentixol, see: Robertson & Trimble (1981 ▶); Valle-Jones & Swarbrick (1981 ▶). For related structures, see: Jones *et al.* (1977 ▶); Post *et al.* (1975*a* ▶,*b* ▶); Siddegowda *et al.* (2011*a* ▶,*b* ▶). For standard bond lengths, see: Allen *et al.* (1987 ▶). For puckering parameters, see: Cremer & Pople (1975 ▶).
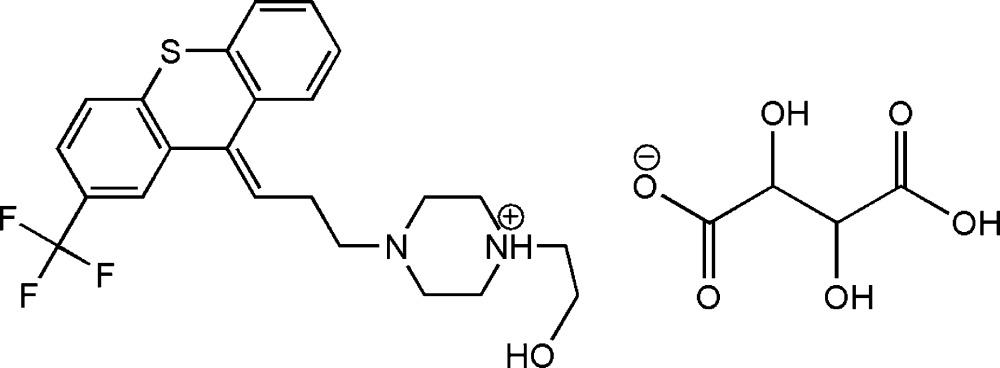


## Experimental   

### 

#### Crystal data  

C_23_H_26_F_3_N_2_OS^+^·C_4_H_5_O_6_^−^
*M* *_r_* = 584.60
Monoclinic, 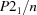

*a* = 9.9239 (3) Å
*b* = 9.1968 (3) Å
*c* = 30.0099 (8) Å
β = 96.617 (3)°
*V* = 2720.68 (13) Å^3^
*Z* = 4
Cu *K*α radiation
μ = 1.67 mm^−1^
*T* = 173 K
0.26 × 0.14 × 0.08 mm

#### Data collection  

Agilent Gemini EOS diffractometer
Absorption correction: multi-scan (*CrysAlis PRO* and *CrysAlis RED*; Agilent, 2012 ▶). *T*_min_ = 0.871, *T*_max_ = 1.000
16827 measured reflections
5325 independent reflections
4331 reflections with *I* > 2σ(*I*)
*R*_int_ = 0.036

#### Refinement  

*R*[*F* ^2^ > 2σ(*F* ^2^)] = 0.078
*wR*(*F* ^2^) = 0.206
*S* = 1.09
5325 reflections
399 parameters
H atoms treated by a mixture of independent and constrained refinement
Δρ_max_ = 0.66 e Å^−3^
Δρ_min_ = −0.71 e Å^−3^

### 

Data collection: *CrysAlis PRO* (Agilent, 2012 ▶); cell refinement: *CrysAlis PRO*; data reduction: *CrysAlis RED* (Agilent, 2012 ▶); program(s) used to solve structure: *SUPERFLIP* (Palatinus & Chapuis, 2007 ▶); program(s) used to refine structure: *SHELXL2012* (Sheldrick, 2008 ▶); molecular graphics: *OLEX2* (Dolomanov *et al.*, 2009 ▶); software used to prepare material for publication: *OLEX2*.

## Supplementary Material

Crystal structure: contains datablock(s) I. DOI: 10.1107/S1600536814001536/su2691sup1.cif

Structure factors: contains datablock(s) I. DOI: 10.1107/S1600536814001536/su2691Isup2.hkl

Supporting information file. DOI: 10.1107/S1600536814001536/su2691Isup3.cml

CCDC reference:

Additional supporting information:  crystallographic information; 3D view; checkCIF report

## Figures and Tables

**Table 1:** Hydrogen-bond geometry (Å, °)

*D*—H⋯*A*	*D*—H	H⋯*A*	*D*⋯*A*	*D*—H⋯*A*
O1*A*—H1*A*⋯O1*B* ^i^	0.82	1.83	2.652 (4)	178
N2*A*—H2*A*⋯O2*B* ^i^	0.96 (4)	1.73 (4)	2.675 (3)	165 (3)
O3*B*—H3*B*⋯O5*B* ^ii^	0.82	2.18	2.903 (3)	147
O4*B*—H4*B*⋯O3*B* ^ii^	0.82	2.14	2.954 (4)	175
O6*B*—H6*B*⋯N1*A*	0.82	1.83	2.629 (4)	165
C3*A*—H3*AB*⋯O5*B*	0.97	2.59	3.314 (4)	132
C5*A*—H5*AA*⋯O2*B* ^iii^	0.97	2.53	3.466 (4)	163
C15*A*—H15*A*⋯O1*A* ^iv^	0.93	2.58	3.397 (5)	148

Symmetry codes: (i) 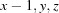; (ii) 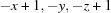; (iii) 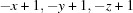; (iv) 
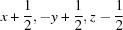
.

**Fig. 1.**
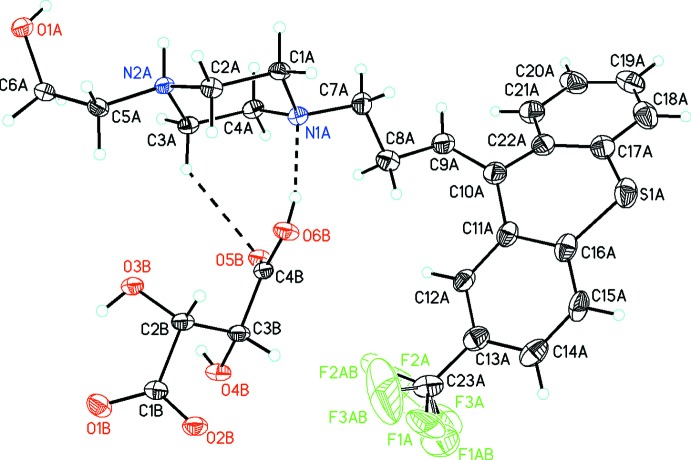
A view of the molecular structure of the title salt, with atom labelling. The displacement ellipsoids are drawn at the 30% probability level. Hydrogen bonds are shown as dashed lines (see Table 1 for details).

**Fig. 2.**
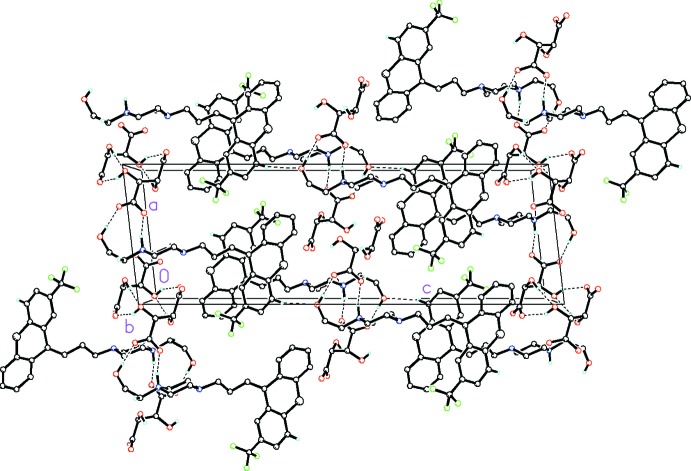
A view along the b axis of the crystal packing of the title compound showing the R22(9) graph set ring motifs involving cations and anions. The hydrogen bonds are shown as dashed lines (see Table 1 for details; H atoms not involved in hydrogen bonding have been omitted for clarity).

**Fig. 3.**
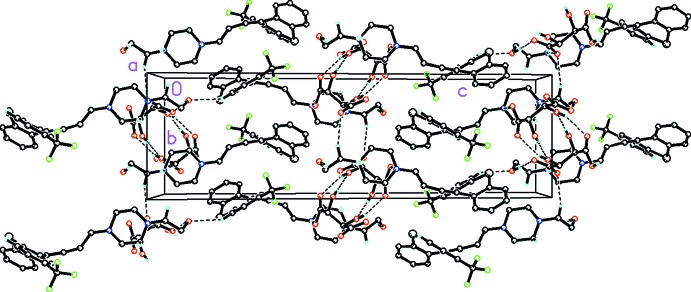
A view along the a axis of the crystal packing of the title compound showing the R22(7), R22(11), R22(10) and R22(12) graph set ring motifs involving the anions. The hydrogen bonds are shown as dashed lines (see Table 1 for details; H atoms not involved in hydrogen bonding have been omitted for clarity).

## supplementary crystallographic information

### 1. Comment

Flupentixol [systematic name: 2-[4-[3-[(EZ)-2-(trifluoromethyl)-9H- thioxanthen-9-ylidene] propyl]piperazin-1-yl]ethanol is a well documented antipsychotic drug of the thioxanthene class. In addition to pure drug preparations, it is also available as deanxit, a combination product containing both melitracen and flupentixol. Low-dose neuroleptics have been applied increasingly in recent years to treat anxiety and depression (Robertson & Trimble, 1981; Valle-Jones & Swarbrick, 1981). The crystal structures of α-flupentixol (Post *et al.*, 1975*b*), β-flupentixol (Post *et al.*, 1975*a*), piflutixol (Jones *et al.*, 1977) have been reported. The crystal structures of he dihydrochloride and difumarate salt of flupentixol has been reported by our group (Siddegowda *et al.*, 2011*a*,*b*). In view of the importance of flupentixol, we prepared the tartrate salt of flupentixol and report herein on its crystal structure.

The title salt, Fig. 1, crystallizes with one independent monocation (A) and monoanion (B) in the asymmetric unit. Bond lengths are in normal ranges (Allen *et al.*, 1987). The monoprotonated piperazine ring in A adopts a slightly disordered chair conformation while the thiopyran ring of the thioxanthene group has a boat conformation. The puckering parameters (Cremer & Pople, 1975) for the various rings are: (N1A//N2A/C1A-C4A) Q, θ, and φ = 0.578 (3) Å, 174.4 (3)° and 192 (3)°, respectively; (S1A/C16A/C11A/C10A/C22A/C17A) Q, θ, and φ = 0.486 (4) Å, 90.7 (5)° and 2.0 (5)°, respectively. The dihedral angle between the mean planes of the two outer aromatic rings of the thioxanthene groups is 31.6 (2)°.

In the crystal, the cations and anions are linked via O-H···O, N-H···O, O-H···N and C-H···O hydrogen bonds (Fig. 1), forming one-dimensional chains propagating along [1 0 0] (see Table 1 and Fig. 2). In addition, R^2^_2_(7), R^2^_2_(11), R^2^_2_(10) and R^2^_2_(12) graph set ring motifs involving the anions (Fig. 3) and R^2^_2_(9) graph set ring motifs involving both the cations and anions (Fig. 2) are observed.

### 2. Experimental

A gift sample of flupentixol was donated by R. L. Fine Chemicals. The title salt was prepared by mixing flupentixol (0.2 g, 4.602 mmol) and tartaric acid (0.07 g, 4.602 mmol) dissolved in 5 mL of dimethyl formamide. The mixture was stirred at 320 K for 30 minutes. X-ray quality colourless block-like crystals were obtained on slow evaporation of the reaction mixture (M.p: 468-474 K).

### 3. Refinement

The NH H atom was located in a difference Fourier map and freely refined. All of the other H atoms were placed in calculated positions and treated as riding atoms: O-H = 0.82 Å, C-H = 0.93, 0.97 and 0.98 Å for CH, methylene and methine H atoms, respectively, with U_iso_(H) = 1.5U_eq_(O), and = 1.2U_eq_(C) for other H atoms. Disorder of the three F atoms of the trifluoromethyl group was modeled over two sets of sites: atoms F1A/F1AB, F2A/F2AB and F3A/F3AB with occupancy ratios of 0.54 (6):0.46 (6), 0.72 (2):0.28 (2) and 0.67 (3):0.33 (3), respectively. 6 reflections with were omitted in the final cycles of refinement.

### Crystal data

**Table:**

C_23_H_26_F_3_N_2_OS^+^·C_4_H_5_O_6_^−^	*F*(000) = 1224
*M**_r_* = 584.60	*D*_x_ = 1.427 Mg m^−^^3^
Monoclinic, *P*2_1_/*n*	Cu *K*α radiation, λ = 1.54184 Å
*a* = 9.9239 (3) Å	Cell parameters from 5128 reflections
*b* = 9.1968 (3) Å	θ = 3.0–72.5°
*c* = 30.0099 (8) Å	µ = 1.67 mm^−^^1^
β = 96.617 (3)°	*T* = 173 K
*V* = 2720.68 (13) Å^3^	Block, colourless
*Z* = 4	0.26 × 0.14 × 0.08 mm

### Data collection

**Table:**

Agilent Gemini EOS diffractometer	5325 independent reflections
Radiation source: Enhance (Cu) X-ray Source	4331 reflections with *I* > 2σ(*I*)
Detector resolution: 16.0416 pixels mm^-1^	*R*_int_ = 0.036
ω scans	θ_max_ = 72.6°, θ_min_ = 3.0°
Absorption correction: multi-scan (*CrysAlis PRO* and *CrysAlis RED*; Agilent, 2012).	*h* = −11→12
*T*_min_ = 0.871, *T*_max_ = 1.000	*k* = −11→11
16827 measured reflections	*l* = −37→25

### Refinement

**Table:**

Refinement on *F*^2^	Primary atom site location: structure-invariant direct methods
Least-squares matrix: full	Hydrogen site location: mixed
*R*[*F*^2^ > 2σ(*F*^2^)] = 0.078	H atoms treated by a mixture of independent and constrained refinement
*wR*(*F*^2^) = 0.206	*w* = 1/[σ^2^(*F*_o_^2^) + (0.0775*P*)^2^ + 5.4936*P*] where *P* = (*F*_o_^2^ + 2*F*_c_^2^)/3
*S* = 1.09	(Δ/σ)_max_ < 0.001
5325 reflections	Δρ_max_ = 0.66 e Å^−^^3^
399 parameters	Δρ_min_ = −0.71 e Å^−^^3^
0 restraints	

### Special details

**Table:**

Geometry. All esds (except the esd in the dihedral angle between two l.s. planes) are estimated using the full covariance matrix. The cell esds are taken into account individually in the estimation of esds in distances, angles and torsion angles; correlations between esds in cell parameters are only used when they are defined by crystal symmetry. An approximate (isotropic) treatment of cell esds is used for estimating esds involving l.s. planes.

### Fractional atomic coordinates and isotropic or equivalent isotropic displacement parameters (Å^2^)

**Table:**

	*x*	*y*	*z*	*U*_iso_*/*U*_eq_	Occ. (<1)
S1A	0.22263 (14)	0.19267 (13)	0.15278 (4)	0.0582 (3)	
F1A	0.7407 (15)	0.0331 (16)	0.3035 (8)	0.097 (7)	0.54 (6)
F1AB	0.758 (2)	−0.042 (12)	0.2900 (16)	0.21 (2)	0.46 (6)
F2A	0.5941 (6)	−0.0768 (15)	0.3334 (2)	0.095 (4)	0.72 (2)
F2AB	0.676 (7)	0.033 (5)	0.3316 (9)	0.23 (3)	0.28 (2)
F3A	0.6844 (14)	−0.1770 (13)	0.2809 (4)	0.128 (6)	0.67 (3)
F3AB	0.624 (4)	−0.182 (4)	0.3087 (18)	0.23 (3)	0.33 (3)
O1A	0.0148 (2)	0.2962 (3)	0.58260 (9)	0.0404 (6)	
H1A	−0.0533	0.2684	0.5668	0.061*	
N1A	0.1313 (3)	0.2330 (3)	0.39016 (9)	0.0307 (6)	
N2A	0.1121 (3)	0.2869 (3)	0.48473 (9)	0.0265 (6)	
H2A	0.015 (4)	0.289 (4)	0.4845 (12)	0.036 (10)*	
C1A	0.0891 (3)	0.3761 (4)	0.40584 (11)	0.0306 (7)	
H1AA	−0.0090	0.3796	0.4045	0.037*	
H1AB	0.1177	0.4519	0.3865	0.037*	
C2A	0.1518 (3)	0.4018 (4)	0.45352 (11)	0.0309 (7)	
H2AA	0.2498	0.4033	0.4544	0.037*	
H2AB	0.1232	0.4960	0.4635	0.037*	
C3A	0.1422 (3)	0.1394 (4)	0.46724 (11)	0.0298 (7)	
H3AA	0.1058	0.0658	0.4857	0.036*	
H3AB	0.2396	0.1260	0.4693	0.036*	
C4A	0.0820 (3)	0.1199 (4)	0.41933 (12)	0.0321 (7)	
H4AA	0.1063	0.0247	0.4088	0.039*	
H4AB	−0.0161	0.1248	0.4175	0.039*	
C5A	0.1858 (3)	0.3134 (4)	0.53071 (11)	0.0319 (7)	
H5AA	0.1816	0.4164	0.5373	0.038*	
H5AB	0.2805	0.2882	0.5303	0.038*	
C6A	0.1307 (3)	0.2288 (4)	0.56834 (12)	0.0339 (8)	
H6AA	0.1073	0.1311	0.5580	0.041*	
H6AB	0.2008	0.2213	0.5936	0.041*	
C7A	0.0795 (4)	0.2111 (4)	0.34276 (12)	0.0389 (8)	
H7AA	0.0832	0.3022	0.3267	0.047*	
H7AB	−0.0145	0.1801	0.3405	0.047*	
C8A	0.1644 (4)	0.0949 (5)	0.32109 (14)	0.0486 (10)	
H8AA	0.2542	0.1330	0.3183	0.058*	
H8AB	0.1744	0.0092	0.3400	0.058*	
C9A	0.0966 (4)	0.0553 (5)	0.27644 (14)	0.0471 (10)	
H9A	0.0035	0.0405	0.2749	0.057*	
C10A	0.1497 (4)	0.0376 (4)	0.23774 (14)	0.0458 (9)	
C11A	0.2971 (4)	0.0496 (4)	0.23310 (13)	0.0431 (9)	
C12A	0.3973 (4)	−0.0074 (4)	0.26503 (14)	0.0434 (9)	
H12A	0.3719	−0.0572	0.2897	0.052*	
C13A	0.5340 (5)	0.0093 (5)	0.26043 (15)	0.0508 (10)	
C14A	0.5736 (5)	0.0809 (5)	0.22360 (16)	0.0565 (12)	
H14A	0.6652	0.0898	0.2201	0.068*	
C15A	0.4755 (5)	0.1393 (5)	0.19180 (14)	0.0547 (11)	
H15A	0.5020	0.1916	0.1677	0.066*	
C16A	0.3389 (5)	0.1206 (4)	0.19556 (14)	0.0482 (10)	
C17A	0.0773 (5)	0.0805 (5)	0.15652 (15)	0.0533 (11)	
C18A	−0.0161 (6)	0.0691 (6)	0.11939 (16)	0.0668 (14)	
H18A	−0.0022	0.1189	0.0934	0.080*	
C19A	−0.1312 (5)	−0.0157 (6)	0.12010 (18)	0.0630 (13)	
H19A	−0.1958	−0.0211	0.0951	0.076*	
C20A	−0.1479 (5)	−0.0926 (6)	0.15899 (17)	0.0633 (13)	
H20A	−0.2229	−0.1529	0.1596	0.076*	
C21A	−0.0565 (5)	−0.0809 (5)	0.19603 (16)	0.0550 (11)	
H21A	−0.0698	−0.1337	0.2216	0.066*	
C22A	0.0591 (4)	0.0105 (5)	0.19664 (14)	0.0472 (10)	
C23A	0.6367 (5)	−0.0546 (6)	0.2954 (2)	0.0642 (13)	
O1B	0.7933 (3)	0.2013 (3)	0.53327 (9)	0.0420 (6)	
O2B	0.8419 (2)	0.3109 (3)	0.47059 (8)	0.0353 (6)	
O3B	0.5331 (2)	0.1935 (3)	0.50950 (8)	0.0350 (6)	
H3B	0.5829	0.1594	0.5306	0.052*	
O4B	0.6567 (2)	−0.0008 (3)	0.44961 (9)	0.0382 (6)	
H4B	0.5999	−0.0500	0.4605	0.057*	
O5B	0.3929 (2)	0.0037 (3)	0.41614 (9)	0.0374 (6)	
O6B	0.3977 (2)	0.2427 (3)	0.40286 (9)	0.0384 (6)	
H6B	0.3165	0.2310	0.3950	0.058*	
C1B	0.7628 (3)	0.2518 (4)	0.49518 (12)	0.0301 (7)	
C2B	0.6127 (3)	0.2418 (4)	0.47601 (11)	0.0301 (7)	
H2B	0.5809	0.3379	0.4653	0.036*	
C3B	0.6004 (3)	0.1351 (4)	0.43639 (12)	0.0321 (7)	
H3BA	0.6511	0.1749	0.4130	0.038*	
C4B	0.4522 (3)	0.1186 (4)	0.41685 (11)	0.0298 (7)	

### Atomic displacement parameters (Å^2^)

**Table:**

	*U*^11^	*U*^22^	*U*^33^	*U*^12^	*U*^13^	*U*^23^
S1A	0.0820 (8)	0.0475 (6)	0.0469 (6)	0.0068 (6)	0.0150 (6)	0.0075 (5)
F1A	0.042 (6)	0.115 (11)	0.126 (12)	−0.029 (5)	−0.030 (7)	0.029 (7)
F1AB	0.073 (8)	0.39 (6)	0.153 (19)	0.00 (2)	0.024 (11)	0.11 (3)
F2A	0.044 (3)	0.173 (10)	0.069 (4)	0.013 (4)	0.004 (2)	0.046 (5)
F2AB	0.40 (7)	0.18 (3)	0.077 (15)	0.12 (4)	−0.04 (3)	−0.069 (18)
F3A	0.129 (10)	0.099 (8)	0.146 (8)	0.077 (7)	−0.023 (6)	−0.013 (5)
F3AB	0.15 (2)	0.19 (3)	0.32 (5)	−0.12 (2)	−0.10 (3)	0.19 (3)
O1A	0.0287 (12)	0.0545 (16)	0.0379 (14)	0.0055 (12)	0.0034 (10)	−0.0116 (12)
N1A	0.0282 (14)	0.0325 (15)	0.0319 (15)	0.0036 (11)	0.0057 (11)	−0.0002 (12)
N2A	0.0212 (13)	0.0293 (14)	0.0296 (14)	0.0016 (11)	0.0049 (11)	−0.0001 (11)
C1A	0.0266 (16)	0.0294 (17)	0.0361 (18)	0.0044 (13)	0.0049 (14)	0.0042 (14)
C2A	0.0289 (16)	0.0277 (16)	0.0360 (18)	−0.0007 (13)	0.0031 (14)	0.0017 (13)
C3A	0.0275 (16)	0.0265 (16)	0.0360 (18)	0.0035 (13)	0.0068 (14)	0.0004 (13)
C4A	0.0299 (17)	0.0294 (17)	0.0379 (19)	0.0004 (14)	0.0076 (14)	−0.0028 (14)
C5A	0.0258 (16)	0.0356 (18)	0.0328 (18)	0.0039 (14)	−0.0026 (13)	−0.0028 (14)
C6A	0.0267 (16)	0.0407 (19)	0.0336 (18)	0.0070 (14)	0.0002 (14)	0.0012 (15)
C7A	0.0389 (19)	0.046 (2)	0.0319 (19)	−0.0005 (16)	0.0038 (15)	−0.0006 (16)
C8A	0.036 (2)	0.062 (3)	0.046 (2)	0.0048 (19)	0.0005 (17)	−0.001 (2)
C9A	0.037 (2)	0.060 (3)	0.045 (2)	0.0037 (19)	0.0045 (17)	−0.0053 (19)
C10A	0.049 (2)	0.042 (2)	0.047 (2)	0.0075 (18)	0.0093 (19)	0.0000 (17)
C11A	0.055 (2)	0.038 (2)	0.038 (2)	−0.0011 (18)	0.0142 (18)	−0.0053 (16)
C12A	0.048 (2)	0.044 (2)	0.041 (2)	0.0052 (18)	0.0178 (18)	−0.0004 (17)
C13A	0.050 (2)	0.050 (2)	0.054 (3)	−0.0012 (19)	0.013 (2)	−0.010 (2)
C14A	0.058 (3)	0.052 (3)	0.065 (3)	−0.016 (2)	0.028 (2)	−0.013 (2)
C15A	0.066 (3)	0.062 (3)	0.038 (2)	−0.020 (2)	0.016 (2)	0.008 (2)
C16A	0.070 (3)	0.036 (2)	0.041 (2)	−0.0008 (19)	0.017 (2)	−0.0062 (17)
C17A	0.068 (3)	0.043 (2)	0.050 (2)	0.022 (2)	0.010 (2)	−0.0046 (19)
C18A	0.092 (4)	0.058 (3)	0.048 (3)	0.030 (3)	0.000 (3)	−0.003 (2)
C19A	0.055 (3)	0.057 (3)	0.072 (3)	0.016 (2)	−0.012 (2)	−0.010 (2)
C20A	0.050 (3)	0.073 (3)	0.063 (3)	0.010 (2)	−0.013 (2)	−0.018 (3)
C21A	0.052 (3)	0.052 (3)	0.060 (3)	0.005 (2)	0.007 (2)	−0.004 (2)
C22A	0.050 (2)	0.046 (2)	0.046 (2)	0.0109 (19)	0.0073 (19)	−0.0062 (18)
C23A	0.041 (2)	0.073 (3)	0.081 (4)	0.002 (2)	0.013 (2)	−0.002 (3)
O1B	0.0320 (13)	0.0488 (15)	0.0436 (15)	−0.0057 (11)	−0.0028 (11)	0.0103 (12)
O2B	0.0216 (11)	0.0418 (14)	0.0427 (14)	−0.0011 (10)	0.0039 (10)	0.0017 (11)
O3B	0.0229 (11)	0.0455 (14)	0.0373 (14)	0.0031 (10)	0.0068 (10)	0.0041 (11)
O4B	0.0249 (11)	0.0362 (13)	0.0528 (16)	0.0069 (10)	0.0020 (11)	−0.0016 (11)
O5B	0.0263 (12)	0.0380 (14)	0.0467 (15)	0.0008 (10)	−0.0003 (10)	0.0023 (11)
O6B	0.0220 (11)	0.0409 (14)	0.0513 (16)	0.0007 (10)	0.0004 (11)	0.0106 (12)
C1B	0.0237 (15)	0.0280 (16)	0.0383 (19)	0.0008 (13)	0.0019 (14)	−0.0033 (14)
C2B	0.0209 (15)	0.0321 (17)	0.0378 (19)	0.0022 (13)	0.0051 (13)	0.0035 (14)
C3B	0.0200 (15)	0.0375 (18)	0.0388 (19)	0.0031 (13)	0.0039 (13)	0.0013 (15)
C4B	0.0224 (15)	0.0373 (18)	0.0304 (17)	0.0037 (14)	0.0058 (13)	0.0037 (14)

### Geometric parameters (Å, º)

**Table:**

S1A—C16A	1.754 (5)	C9A—H9A	0.9300
S1A—C17A	1.788 (5)	C9A—C10A	1.340 (6)
F1A—C23A	1.311 (12)	C10A—C11A	1.489 (6)
F1AB—F2AB	1.71 (8)	C10A—C22A	1.461 (6)
F1AB—C23A	1.24 (2)	C11A—C12A	1.401 (6)
F2A—C23A	1.278 (8)	C11A—C16A	1.406 (6)
F2AB—C23A	1.37 (3)	C12A—H12A	0.9300
F3A—C23A	1.314 (8)	C12A—C13A	1.387 (6)
F3AB—C23A	1.25 (2)	C13A—C14A	1.382 (6)
O1A—H1A	0.8200	C13A—C23A	1.497 (7)
O1A—C6A	1.415 (4)	C14A—H14A	0.9300
N1A—C1A	1.474 (4)	C14A—C15A	1.391 (7)
N1A—C4A	1.479 (4)	C15A—H15A	0.9300
N1A—C7A	1.470 (4)	C15A—C16A	1.384 (6)
N2A—H2A	0.96 (4)	C17A—C18A	1.369 (7)
N2A—C2A	1.495 (4)	C17A—C22A	1.395 (6)
N2A—C3A	1.497 (4)	C18A—H18A	0.9300
N2A—C5A	1.505 (4)	C18A—C19A	1.385 (8)
C1A—H1AA	0.9700	C19A—H19A	0.9300
C1A—H1AB	0.9700	C19A—C20A	1.391 (8)
C1A—C2A	1.512 (5)	C20A—H20A	0.9300
C2A—H2AA	0.9700	C20A—C21A	1.356 (6)
C2A—H2AB	0.9700	C21A—H21A	0.9300
C3A—H3AA	0.9700	C21A—C22A	1.421 (6)
C3A—H3AB	0.9700	O1B—C1B	1.239 (4)
C3A—C4A	1.503 (5)	O2B—C1B	1.261 (4)
C4A—H4AA	0.9700	O3B—H3B	0.8200
C4A—H4AB	0.9700	O3B—C2B	1.419 (4)
C5A—H5AA	0.9700	O4B—H4B	0.8200
C5A—H5AB	0.9700	O4B—C3B	1.407 (4)
C5A—C6A	1.524 (5)	O5B—C4B	1.208 (4)
C6A—H6AA	0.9700	O6B—H6B	0.8200
C6A—H6AB	0.9700	O6B—C4B	1.312 (4)
C7A—H7AA	0.9700	C1B—C2B	1.537 (4)
C7A—H7AB	0.9700	C2B—H2B	0.9800
C7A—C8A	1.549 (6)	C2B—C3B	1.535 (5)
C8A—H8AA	0.9700	C3B—H3BA	0.9800
C8A—H8AB	0.9700	C3B—C4B	1.526 (4)
C8A—C9A	1.474 (6)		
			
C16A—S1A—C17A	101.7 (2)	C12A—C11A—C16A	118.0 (4)
C23A—F1AB—F2AB	52 (3)	C16A—C11A—C10A	119.6 (4)
C23A—F2AB—F1AB	46 (2)	C11A—C12A—H12A	119.5
C6A—O1A—H1A	109.5	C13A—C12A—C11A	121.1 (4)
C1A—N1A—C4A	108.3 (2)	C13A—C12A—H12A	119.5
C7A—N1A—C1A	110.6 (3)	C12A—C13A—C23A	118.8 (4)
C7A—N1A—C4A	111.9 (3)	C14A—C13A—C12A	120.3 (4)
C2A—N2A—H2A	108 (2)	C14A—C13A—C23A	120.9 (4)
C2A—N2A—C3A	110.1 (2)	C13A—C14A—H14A	120.3
C2A—N2A—C5A	108.9 (3)	C13A—C14A—C15A	119.4 (4)
C3A—N2A—H2A	105 (2)	C15A—C14A—H14A	120.3
C3A—N2A—C5A	111.9 (3)	C14A—C15A—H15A	119.6
C5A—N2A—H2A	112 (2)	C16A—C15A—C14A	120.7 (4)
N1A—C1A—H1AA	109.7	C16A—C15A—H15A	119.6
N1A—C1A—H1AB	109.7	C11A—C16A—S1A	122.1 (4)
N1A—C1A—C2A	109.9 (3)	C15A—C16A—S1A	117.5 (3)
H1AA—C1A—H1AB	108.2	C15A—C16A—C11A	120.4 (4)
C2A—C1A—H1AA	109.7	C18A—C17A—S1A	118.0 (4)
C2A—C1A—H1AB	109.7	C18A—C17A—C22A	121.6 (5)
N2A—C2A—C1A	111.9 (3)	C22A—C17A—S1A	120.4 (4)
N2A—C2A—H2AA	109.2	C17A—C18A—H18A	119.6
N2A—C2A—H2AB	109.2	C17A—C18A—C19A	120.8 (5)
C1A—C2A—H2AA	109.2	C19A—C18A—H18A	119.6
C1A—C2A—H2AB	109.2	C18A—C19A—H19A	120.7
H2AA—C2A—H2AB	107.9	C18A—C19A—C20A	118.5 (5)
N2A—C3A—H3AA	109.3	C20A—C19A—H19A	120.7
N2A—C3A—H3AB	109.3	C19A—C20A—H20A	119.5
N2A—C3A—C4A	111.8 (3)	C21A—C20A—C19A	120.9 (5)
H3AA—C3A—H3AB	107.9	C21A—C20A—H20A	119.5
C4A—C3A—H3AA	109.3	C20A—C21A—H21A	119.3
C4A—C3A—H3AB	109.3	C20A—C21A—C22A	121.4 (5)
N1A—C4A—C3A	111.1 (3)	C22A—C21A—H21A	119.3
N1A—C4A—H4AA	109.4	C17A—C22A—C10A	121.5 (4)
N1A—C4A—H4AB	109.4	C17A—C22A—C21A	116.5 (4)
C3A—C4A—H4AA	109.4	C21A—C22A—C10A	121.9 (4)
C3A—C4A—H4AB	109.4	F1A—C23A—F3A	106.4 (11)
H4AA—C4A—H4AB	108.0	F1A—C23A—C13A	110.3 (8)
N2A—C5A—H5AA	108.6	F1AB—C23A—F2AB	82 (5)
N2A—C5A—H5AB	108.6	F1AB—C23A—F3AB	105 (4)
N2A—C5A—C6A	114.7 (3)	F1AB—C23A—C13A	117.8 (14)
H5AA—C5A—H5AB	107.6	F2A—C23A—F1A	105.5 (12)
C6A—C5A—H5AA	108.6	F2A—C23A—F3A	109.1 (9)
C6A—C5A—H5AB	108.6	F2A—C23A—C13A	114.9 (4)
O1A—C6A—C5A	112.0 (3)	F2AB—C23A—C13A	115.7 (12)
O1A—C6A—H6AA	109.2	F3A—C23A—C13A	110.3 (6)
O1A—C6A—H6AB	109.2	F3AB—C23A—F2AB	109 (3)
C5A—C6A—H6AA	109.2	F3AB—C23A—C13A	120.3 (11)
C5A—C6A—H6AB	109.2	C2B—O3B—H3B	109.5
H6AA—C6A—H6AB	107.9	C3B—O4B—H4B	109.5
N1A—C7A—H7AA	109.5	C4B—O6B—H6B	109.5
N1A—C7A—H7AB	109.5	O1B—C1B—O2B	126.8 (3)
N1A—C7A—C8A	110.8 (3)	O1B—C1B—C2B	116.5 (3)
H7AA—C7A—H7AB	108.1	O2B—C1B—C2B	116.7 (3)
C8A—C7A—H7AA	109.5	O3B—C2B—C1B	110.4 (3)
C8A—C7A—H7AB	109.5	O3B—C2B—H2B	109.3
C7A—C8A—H8AA	109.7	O3B—C2B—C3B	110.3 (3)
C7A—C8A—H8AB	109.7	C1B—C2B—H2B	109.3
H8AA—C8A—H8AB	108.2	C3B—C2B—C1B	108.4 (3)
C9A—C8A—C7A	109.8 (3)	C3B—C2B—H2B	109.3
C9A—C8A—H8AA	109.7	O4B—C3B—C2B	110.8 (3)
C9A—C8A—H8AB	109.7	O4B—C3B—H3BA	108.3
C8A—C9A—H9A	115.3	O4B—C3B—C4B	110.7 (3)
C10A—C9A—C8A	129.4 (4)	C2B—C3B—H3BA	108.3
C10A—C9A—H9A	115.3	C4B—C3B—C2B	110.4 (3)
C9A—C10A—C11A	124.1 (4)	C4B—C3B—H3BA	108.3
C9A—C10A—C22A	119.2 (4)	O5B—C4B—O6B	125.0 (3)
C22A—C10A—C11A	116.7 (4)	O5B—C4B—C3B	122.7 (3)
C12A—C11A—C10A	122.4 (4)	O6B—C4B—C3B	112.3 (3)
			
S1A—C17A—C18A—C19A	−179.8 (4)	C12A—C13A—C14A—C15A	1.7 (7)
S1A—C17A—C22A—C10A	6.3 (5)	C12A—C13A—C23A—F1A	−141.2 (14)
S1A—C17A—C22A—C21A	−177.6 (3)	C12A—C13A—C23A—F1AB	178 (6)
F1AB—F2AB—C23A—F1A	−22 (3)	C12A—C13A—C23A—F2A	−22.2 (10)
F1AB—F2AB—C23A—F2A	138 (3)	C12A—C13A—C23A—F2AB	−88 (4)
F1AB—F2AB—C23A—F3A	51 (4)	C12A—C13A—C23A—F3A	101.6 (10)
F1AB—F2AB—C23A—F3AB	104 (4)	C12A—C13A—C23A—F3AB	47 (4)
F1AB—F2AB—C23A—C13A	−117 (3)	C13A—C14A—C15A—C16A	−3.1 (7)
F2AB—F1AB—C23A—F1A	27 (3)	C14A—C13A—C23A—F1A	40.3 (14)
F2AB—F1AB—C23A—F2A	−43 (4)	C14A—C13A—C23A—F1AB	−1 (6)
F2AB—F1AB—C23A—F3A	−142.6 (18)	C14A—C13A—C23A—F2A	159.3 (8)
F2AB—F1AB—C23A—F3AB	−108 (3)	C14A—C13A—C23A—F2AB	94 (4)
F2AB—F1AB—C23A—C13A	115 (3)	C14A—C13A—C23A—F3A	−77.0 (10)
N1A—C1A—C2A—N2A	−58.7 (3)	C14A—C13A—C23A—F3AB	−132 (4)
N1A—C7A—C8A—C9A	169.7 (3)	C14A—C15A—C16A—S1A	−177.8 (4)
N2A—C3A—C4A—N1A	57.1 (3)	C14A—C15A—C16A—C11A	3.8 (7)
N2A—C5A—C6A—O1A	−80.8 (4)	C16A—S1A—C17A—C18A	−157.3 (3)
C1A—N1A—C4A—C3A	−61.0 (3)	C16A—S1A—C17A—C22A	24.3 (4)
C1A—N1A—C7A—C8A	158.2 (3)	C16A—C11A—C12A—C13A	1.8 (6)
C2A—N2A—C3A—C4A	−51.9 (3)	C17A—S1A—C16A—C11A	−26.5 (4)
C2A—N2A—C5A—C6A	165.7 (3)	C17A—S1A—C16A—C15A	155.1 (4)
C3A—N2A—C2A—C1A	53.0 (3)	C17A—C18A—C19A—C20A	−1.9 (7)
C3A—N2A—C5A—C6A	−72.3 (3)	C18A—C17A—C22A—C10A	−172.0 (4)
C4A—N1A—C1A—C2A	61.3 (3)	C18A—C17A—C22A—C21A	4.1 (6)
C4A—N1A—C7A—C8A	−80.9 (4)	C18A—C19A—C20A—C21A	2.5 (7)
C5A—N2A—C2A—C1A	176.0 (3)	C19A—C20A—C21A—C22A	0.3 (7)
C5A—N2A—C3A—C4A	−173.2 (3)	C20A—C21A—C22A—C10A	172.6 (4)
C7A—N1A—C1A—C2A	−175.7 (3)	C20A—C21A—C22A—C17A	−3.5 (6)
C7A—N1A—C4A—C3A	176.8 (3)	C22A—C10A—C11A—C12A	−141.7 (4)
C7A—C8A—C9A—C10A	135.7 (5)	C22A—C10A—C11A—C16A	38.5 (5)
C8A—C9A—C10A—C11A	2.8 (8)	C22A—C17A—C18A—C19A	−1.5 (7)
C8A—C9A—C10A—C22A	−174.6 (4)	C23A—C13A—C14A—C15A	−179.8 (4)
C9A—C10A—C11A—C12A	40.8 (6)	O1B—C1B—C2B—O3B	8.5 (4)
C9A—C10A—C11A—C16A	−139.0 (4)	O1B—C1B—C2B—C3B	−112.4 (3)
C9A—C10A—C22A—C17A	136.5 (4)	O2B—C1B—C2B—O3B	−171.3 (3)
C9A—C10A—C22A—C21A	−39.4 (6)	O2B—C1B—C2B—C3B	67.8 (4)
C10A—C11A—C12A—C13A	−178.1 (4)	O3B—C2B—C3B—O4B	−65.3 (3)
C10A—C11A—C16A—S1A	−1.6 (5)	O3B—C2B—C3B—C4B	57.7 (4)
C10A—C11A—C16A—C15A	176.7 (4)	O4B—C3B—C4B—O5B	4.4 (5)
C11A—C10A—C22A—C17A	−41.1 (6)	O4B—C3B—C4B—O6B	−178.0 (3)
C11A—C10A—C22A—C21A	143.0 (4)	C1B—C2B—C3B—O4B	55.6 (3)
C11A—C12A—C13A—C14A	−1.1 (6)	C1B—C2B—C3B—C4B	178.6 (3)
C11A—C12A—C13A—C23A	−179.6 (4)	C2B—C3B—C4B—O5B	−118.7 (4)
C12A—C11A—C16A—S1A	178.5 (3)	C2B—C3B—C4B—O6B	58.9 (4)
C12A—C11A—C16A—C15A	−3.1 (6)		

### Hydrogen-bond geometry (Å, º)

**Table:**

*D*—H···*A*	*D*—H	H···*A*	*D*···*A*	*D*—H···*A*
O1*A*—H1*A*···O1*B*^i^	0.82	1.83	2.652 (4)	178
N2*A*—H2*A*···O2*B*^i^	0.96 (4)	1.73 (4)	2.675 (3)	165 (3)
O3*B*—H3*B*···O5*B*^ii^	0.82	2.18	2.903 (3)	147
O4*B*—H4*B*···O3*B*^ii^	0.82	2.14	2.954 (4)	175
O6*B*—H6*B*···N1*A*	0.82	1.83	2.629 (4)	165
C3*A*—H3*AB*···O5*B*	0.97	2.59	3.314 (4)	132
C5*A*—H5*AA*···O2*B*^iii^	0.97	2.53	3.466 (4)	163
C15*A*—H15*A*···O1*A*^iv^	0.93	2.58	3.397 (5)	148

Symmetry codes: (i) *x*−1, *y*, *z*; (ii) −*x*+1, −*y*, −*z*+1; (iii) −*x*+1, −*y*+1, −*z*+1; (iv) *x*+1/2, −*y*+1/2, *z*−1/2.
